# The Impact of Heterogeneity on Single-Cell Sequencing

**DOI:** 10.3389/fgene.2019.00008

**Published:** 2019-03-01

**Authors:** Samantha L. Goldman, Matthew MacKay, Ebrahim Afshinnekoo, Ari M. Melnick, Shuxiu Wu, Christopher E. Mason

**Affiliations:** ^1^Department of Physiology and Biophysics, Weill Cornell Medical College, New York, NY, United States; ^2^The HRH Prince Alwaleed Bin Talal Bin Abdulaziz Alsaud Institute for Computational Biomedicine, Weill Cornell Medicine, New York, NY, United States; ^3^WorldQuant Initiative for Quantitative Prediction, Weill Cornell Medicine, New York, NY, United States; ^4^Department of Medicine, Weill Cornell Medicine, New York, NY, United States; ^5^Hangzhou Cancer Institute, Hangzhou Cancer Hospital, Hangzhou, China; ^6^Department of Radiation Oncology, Hangzhou Cancer Hospital, Hangzhou, China; ^7^The Feil Family Brain and Mind Research Institute, New York, NY, United States

**Keywords:** single-cell sequencing, heterogeneity, scRNA-seq, NGS, RNA, single cells

## Abstract

The importance of diversity and cellular specialization is clear for many reasons, from population-level diversification, to improved resiliency to unforeseen stresses, to unique functions within metazoan organisms during development and differentiation. However, the level of cellular heterogeneity is just now becoming clear through the integration of genome-wide analyses and more cost effective Next Generation Sequencing (NGS). With easy access to single-cell NGS (scNGS), new opportunities exist to examine different levels of gene expression and somatic mutational heterogeneity, but these assays can generate yottabyte scale data. Here, we model the importance of heterogeneity for large-scale analysis of scNGS data, with a focus on the utilization in oncology and other diseases, providing a guide to aid in sample size and experimental design.

## Introduction

It has been well-documented, both theoretically (Elsasser, 1984) and experimentally, that nearly all cellular systems are heterogenous (Altschuler and Wu, 2010). Heterogeneity may arise for a number of different reasons, and at many different levels, in order to improve survival and functionality. Both single-celled and multicellular organisms employ population-level survival strategies such as bet-hedging in order to achieve a better chance of survival when faced with new stresses though having a diverse population (Grimbergen et al., 2015). At a single-organism level, diversity further enables the existence of specialization and, within metazoan organisms, differentiation (Hadjantonakis and Arias, 2016).

Cellular heterogeneity can be measured in several different ways, most commonly via genomic, epigenomic, transcriptomic, and proteomic studies. However, the level of heterogeneity at one level of expression or regulation may not be the same at another level. Cells within a given person have nearly identical genomes, yet through specific modifications throughout development and disease, may generate many distinct cell types with unique expression profiles. Even the genome itself may be specifically rewired to generate increased genetic diversity within specific cell types, most notably B- and T-cells through V(D)J recombination. Uncovering the true diversity of cells is crucial to better understand cellular communication and responsibility within both healthy and disease states. It is now well understood that differentiation throughout development allows for the necessary cellular specialization required for complex multicellular system function. Further, specific epigenomic modifications allow for this precise differentiation which inevitably results in the cascade of cellular diversity present in humans, and also is important in cancer (Li et al., 2014, 2016).

Next generation sequencing (NGS) is continuously being used more and more due to its rapidly decreased costs and ability to generate a large amount of data (Mason et al., 2014), with new data sets even being generated in zero gravity (McIntyre et al., 2016; Castro-Wallace et al., 2017). Within bulk-NGS analyses, many, typically hundreds of thousands to millions, of cells are analyzed at once. This generates an averaged picture of a given population of cells, and thus majority of our understanding of different cell and tissue types comes from the analysis of bulk experimentation which may underestimate the true heterogeneity of cells. Bulk-NGS is simply ill-equipped to address some important questions revolving around cellular heterogeneity. Single-cell NGS (scNGS) attempts to resolve issues facing bulk-NGS through the ability to relate sequences to a given cell, across the genetic, transcriptomic, epigenomic, and proteomic levels. This approach reduces the issue of data generalization which is prevalent in some bulk-NGS studies. However, scNGS is not without its faults. One of the main issues with scNGS is its cost and, though it has considerably decreased in recent years, it is still a large factor when designing experimentations, as well as technical issues and challenges in sensitivity. Here, we will outline the importance of cellular heterogeneity, assess factors of scNGS heterogeneity, and provide a practical sample size guide to aid in experimental design.

## The Importance of Cellular Heterogeneity

Having a heterogeneous (i.e., diverse) population is beneficial for cellular systems for the same reason why it is beneficial for there to be variation among many organisms in a single species – bet-hedging (Beaumont et al., 2009). Bet-hedging is a population-level survival strategy in which less-fit individuals are maintained in a population as a precaution; if the environment were to drastically change, the originally less-fit organisms may be adapted to the new environment, thereby assuring the survival of the population (Grimbergen et al., 2015). In an ever-changing environment, a population has a greater overall fitness if there is greater diversity. In this way, the evolution adaptation of all cellular systems can be modeled in terms of Darwinian evolution.

There are many causes of cellular heterogeneity. Firstly, populations of cells will naturally contain individuals that develop random mutations. These unique subclones can become significant portions of the population if that mutation confers a selective advantage and proliferates. However, not all cellular heterogeneity is genetic. Rather, much heterogeneity is phenotypic, and is frequently expressed in transcriptomes that vary from cell to cell. This heterogeneity can arise via external or internal factors. Extrinsic heterogeneity can lead to phenotypic plasticity in response to an environmental change, and only affects the part of the population that is exposed to the causative environment (Huang, 2009). It can also include variables such as cell-cycle stage and cell size (Singh and Soltani, 2013). Intrinsic heterogeneity is a more nuanced phenomenon, and is a result of stochastic events, such as gene expression noise (Huang, 2009), rather than a changing intracellular environment (Elowitz et al., 2002).

Because of stochastic gene fluctuation, there are varying levels of protein abundance in different cells in a population at any given time. This is most easily visualized via flow cytometry, which yields a bell-shaped curve (Brock et al., 2009). Stochastic gene expression may have its evolutionary advantages, as well. In the same way that populations of cells maintain random mutations in bet-hedging, populations of clonal, unicellular organisms may maintain variation via stochastic gene expression to ensure overall survival (Raj and van Oudenaarden, 2008). Although stochastic gene expression is a significant contributor to heterogeneity, it is not the only cause. The sub-state of any given genome/cell depends on a number of factors, including epigenetics, alternative splicing sites, post-translational modifications, and sometimes even microbial interactions (Shabaan et al., 2018). These processes are not always stochastic, and can therefore lead to “directed” heterogeneity, instead of the more random “non-directed” heterogeneity of stochastic gene expression (Chang and Marshall, 2017).

Interestingly, non-genetic, cellular heterogeneity also plays an important role in development. Early in the developmental process, before the small population of cells is beginning to differentiate, these cells are theoretically identical. However, as the cells begin to differentiate, they display non-genetic heterogeneity. The body of research on the role of heterogeneity in development is largely focused on transcriptional heterogeneity (Griffiths et al., 2018), which is a driver of differentiation of pluripotent stem cells. More recent work has also shown that RNA modifications, called the epitranscriptome (Saletore et al., 2012), can also lead to differential response of human cells to both disease and infection (Gokhale et al., 2016; Vu et al., 2017). Also, some transcriptional sub-states are heritable through several generations of cell divisions. Signaling factors, developmental regulators, and chromatin regulators contribute to transcriptional heterogeneity in stem cells (Kumar et al., 2014). “Directed” heterogeneity has been shown to lead the process behind the development of a body plan in *Drosophila melanogaster* (Chang and Marshall, 2017).

Even after development, all human tissue systems experience some level of differentiation. This allows cells to specialize, leading to a more flexible biological system. This principle has been most notably studied in the nervous and immune systems. In the central nervous system, for instance, there are dozens of different types of neurons. Subsets of these neurons form the myriad different regions within the brain (Emery and Barres, 2008). One phenotypic hallmark of heterogeneity in the nervous system, for example, is the distribution of mitochondria within the neuron. This heterogeneity is exhibited both regionally within the brain (e.g., brain regions that require more energy are composed of neurons with more mitochondria) (Dubinsky, 2009) and within individual neurons. This distribution differs greatly depending on the immediate and current needs of the neuron, and is regulated by a complex system of proteins (Course and Wang, 2016). In the immune system, monocytes, macrophages (Gordon and Taylor, 2005), B-cells, and T-cells show heterogeneity. As an example, T-cell heterogeneity is essential for an effective immune response, since subtle differences in T-cell receptors (TCRs) enable the identification and elimination of foreign invaders (Durlanik and Thiel, 2015). However, in autoimmune disease, faulty TCR diversification can result in the improper identification of “self” as an invader resulting in normal tissue destruction.

Different diseases leverage heterogeneity to their advantage. A “survival of the fittest” model for cellular heterogeneity can be applied not only to populations of single-celled organisms, but also to tumors. Cancer cells continuously acquire and pass down genetic and epigenetic modifications to subsequent generations of cancer cells resulting in heterogeneity. These genetic mutations and epigenetic shifts may further lead to changes in fitness (Li et al., 2016). Cancer cells are often exposed to hostile environments, such as chemotherapy and radiation, during treatment (Afshinnekoo and Mason, 2016). Through bet-hedging, and therefore maintenance of a heterogeneous population, the chance of resistance or relapse from treatment is dramatically increased. As these cancer cells are all in the same small environment and are all competing for the same limited resources, there are complex interactions between different subclones that further reinforce these Darwinian relationships (Tabassum and Polyak, 2015). Cancer cells can be further driven into a “survival of the fittest” scenario via treatment with a chemotherapeutic drug, as this may lead to the selection for cancer-variants that are resistant to the drug. Over time, this could lead to chemotherapeutic resistance within the whole tumor (Dagogo-Jack and Shaw, 2017), as well as tumor sub-types (Shih et al., 2017). Indeed, it has been shown that a single tumor biopsy dramatically underrepresents the genetic diversity present within an entire tumor (Gerlinger et al., 2012). However, heterogeneity is not only clinically relevant in regards to chemotherapy. Immunotherapies can also be profoundly impacted by heterogeneity. Liver cancer-targeted immunotherapy is designed around tumor-infiltrating T-cells. Through the use of single-cell RNA sequencing, 11 tumor-infiltrating T-cell sub-states have been identified. Each of these sub-states has a unique profile of up- and downregulated genes, which may impact the efficacy of any immunotherapies (Zheng et al., 2017).

Intratumoral heterogeneity has been extensively studied through single-cell sequencing methods. For example, single-cell RNA sequencing has revealed significant heterogeneity in primary glioblastomas (Patel et al., 2014). Additionally, increased levels of heterogeneity in these tumors was inversely correlated with survival, indicating that intratumor heterogeneity should be an essential clinical factor, including events from DNA transposition (Henssen et al., 2017). Metastatic melanoma is also highly transcriptionally heterogeneous, and this heterogeneity is multifaceted; it is associated with a number of factors, including cell cycle stage, location, and chemotherapeutic resistance (Tirosh et al., 2016). The use of RNA sequencing here is key, as transcriptomics captures fine details of non-genetic heterogeneity that other sequencing methods may have missed. Shifting of cellular heterogeneity is not just a hallmark of cancer, but of many other diseases, but here we will focus on the relevance for cancer.

## Assessing Heterogeneity

Heterogeneity itself is a gradient which may be based on variable changes in the transcriptome or more permanent changes within the genome. Differences seen between cells may be temporal due to cell-cycle states, or spatial due to external stimuli (Dagogo-Jack and Shaw, 2017). Also, differences between cells may exist at any processing level of the cell, from the genome to transcriptome to proteome, or due to any additional modifications which may exist. With this in mind, it could be possible to define all cells as heterogeneous. However, two disparate cells might not behave functionally different, and their heterogeneity would therefore not be considered impactful (Altschuler and Wu, 2010). The overall assessment of cellular heterogeneity is therefore context-specific and the technologies used to assess cellular differences need to be considered carefully.

Proteomic and cell-marker classification has been historically used to discern cell types. Immunohistochemistry (IHC) can be used to distinguish immune cell types within healthy systems (Reuben et al., 2017b) or even the cancer subtyping such as HER2 expression within breast cancer (Potts et al., 2012). Surface markers help to distinguish cell types into broad classification, but this type of analysis required prior gene expression knowledge and specific antibody usage. Other approaches, such as whole genome sequencing (WGS), bisulfite sequencing, and RNA sequencing, allow for genome-wide analysis (Mason et al., 2017). Historically these techniques are done on heterogeneous tissue samples, generating an averaged picture of the tissue of interest (bulk-NGS). Although bulk-NGS has a tendency to generalize heterogeneity, certain biological understanding and computational modeling can mitigate this effect within genomic and epigenomic analyses.

Bulk-WGS can be directly used to assess the existence of subclonal mutations through the use of variant allele frequencies (VAFs). Through the modeling of VAFs and copy number changes, an understanding of the clonal architecture may be inferred from such bulk-NGS data. One such method, *Canopy*, uses a Bayesian analysis to identify subpopulations and build a phylogenetic tree detailing their likely evolutionary history (Jiang et al., 2016). Long read bulk TCR sequencing can also be used directly to assess clonal structures under the assumption that there is a unique V(D)J recombination per subclone. As such, the quantity of a given TCR gene can be directly related to the abundance of that subclone and the number of different TCR genes relates to the overall heterogeneity and diversity of the T-cell population. TCR sequencing has also been used, and has shown intratumoral heterogeneity in localized lung carcinomas, which may confer post-surgical recurrence (Reuben et al., 2017a). As epigenetics also plays a significant role in heterogeneity, bisulfite sequencing can be used to study patterns of DNA methylation and estimate clonality, such as with the algorithm methclone (Li et al., 2014). Bisulfite sequencing has also been used to reveal heterogeneity in DNA methylation of the *MLH1* (a mismatch repair gene) promoter across several endometrial tumors (Varley et al., 2009).

While many bulk-NGS methods rely on mixture models of the VAFs to analyze small indels and point mutations, these methods often rely on the copy number of the gene in question, which can be altered in cancers, and are unable to relate multiple mutations which exist at low frequencies (Jiang et al., 2016). Additionally, bulk sequencing has a tendency to report what an “average” cell in a population would look like and for that reason would not be usable in the analysis of an all-or-nothing response (Altschuler and Wu, 2010). For example, *Xenopus* oocytes, have a binary response when signaled by progesterone to begin a process of maturation; they either mature or they do not (Ferrell and Machleder, 1998). In this case, looking at an average of two distinct oocyte subpopulations – one that has been signaled to mature and one that has not – would artificially yield a biologically impossible “mean oocyte” that has committed to maturation half-way (Altschuler and Wu, 2010).

There has been a significant effort within the field to quantitatively measure heterogeneity and relate it to a functional change. One approach to this is to quantify stochastic gene expression. This has been done through dividing stochastic gene expression into its intrinsic and extrinsic components via a two-color reporter experiment and deriving analytical formulas to measure each component of noise (Singh and Soltani, 2013). Systems have also been developed to quantify the individual contribution of unique processes to stochastic gene expression, and therefore to heterogeneity. For example, experimentally generated models have been used to quantify the individual contribution to chromatin dynamics in isogenic chicken-cell populations (Viñuelas et al., 2013). Also, shifted gene expression dynamics have been shown to drive cell fate choice for hematopoietic progenitors (Kleppe et al., 2017), induced pluripotent stem cells (iPSCs), and the mouse inner-cell mass during embryogenesis (Mojtahedi et al., 2016; Bargaje et al., 2017; Mohammed et al., 2017).

## Utilization of scNGS

To best understand cellular heterogeneity, single cells must be studied individually through the use of scNGS. Since assessing cellular co-occurrence is the main drawback of bulk-NGS, many studies have also been conducted to further elucidate clonal structures using single-cell DNAseq [including whole exome sequencing (WES) or WGS], bisulfite sequencing, and ATACseq (assay for transposable accessible chromatin, ATAC). Given the variability and importance of gene expression, sc-RNAseq is one of the most used single-cell sequencing techniques (Supplementary Table S1). Single-cell multi-omic analyses are also possible to uncover the true level of heterogeneity across expression levels within cells (Macaulay et al., 2017), which enable examination of the genome, transcriptome, and epigenome at once. scNGS has the ability to resolve noise in bulk-NGS through the additional ability to trace generated reads back to their cell of origin. Though, this added benefit comes at a steep monetary cost, as single-cell sequencing is still much more expensive than more traditional bulk NGS given the need to sequence more (Supplementary Table S2). Also, subpopulations of cancer cells can be found by scATAC-seq, which has the power to identify specific chromatin motifs. Indeed, when combined with RNA-seq, it has been used to identify epigenetic plasticity between two cell subpopulations (Litzenburger et al., 2017).

There are currently dozens of variations of techniques to study the genome, epigenome, transcriptome, and epitranscriptome of cells, and here, we focus on those most commonly in use (Supplementary Table S1). Each of these technologies has had a significant impact on numerous fields, including immunology, oncology, and microbiology. Because the scope of the benefits of single-cell analysis is so wide, there is tremendous pressure to advance the technologies in the field. This is evident in the dramatic increase in recent years in publications referencing single-cell technologies (Wang and Navin, 2015). These techniques are highly varied, from manual manipulation (Pan et al., 2013) to droplet microfluidics used for sc-WGS (Hosokawa et al., 2017) to the creation of an RNA-library (Hedlund and Deng, 2018), such as bisulfite sequencing, can also be used on the single-cell level (Clark et al., 2017). A novel approach that combines Raman spectroscopy with an algorithmic biomolecular component analysis (microRaman-BCA) allows for the profiling of single organelles from a cell. Because this technique does not destroy the cell during analysis, the study can be performed multiple times on the same cell, providing a better picture of heterogeneity over time (Kuzmin et al., 2017).

While much of the current knowledge of cellular heterogeneity is transcriptional, newer techniques such as single-cell epigenomics have tremendous potential to study heterogeneity (Hassan et al., 2017) and may be able to provide further insights into the characterization and mechanisms of heterogeneity (Clark et al., 2016). Several topics in epigenomics are best suited to study with single-cell methods, including the relationship between transcriptional heterogeneity and epigenetic heterogeneity, which may vary greatly from cell to cell. Another application of single-cell sequencing is to study tumor resistance and therapeutic response to decrease the chance of resistance or relapse. scNGS can be used to not only detect heterogenous subclones within a tumor, but also to characterize these cells. Additionally, it can be used to characterize metastases and to create an effective treatment plan that minimizes the chance of chemotherapeutic resistance of specific subclones (Liang and Fu, 2017). In one study, analysis via deep whole-exome sequencing revealed that 75% of relapsed tumors in pediatric B-acute lymphoblastic leukemia were descendants of originally rare subclones (Ma et al., 2015). Given technical and sampling limitations, it is possible that resistant subclones existed within more patients. Although scNGS is currently expensive, treatment for cancer is often much more expensive. For this reason, any possible technique that could lead to a more effective therapy (even an expensive one like scRNA-seq) has clinical potential (Shalek and Benson, 2017).

Additionally, subclones can communicate and interact with each other, leading to complex relationships that may only be fully elucidated via scNGS. Although some of these interactions are neutral, they can also be positive (leading to a commensalistic/mutualistic relationships in which one or both of the subclones benefit), or negative (leading to competition between subclones, e.g.), and can contribute to the chemotherapeutic resistance of one or more subclones within a tumor (Tabassum and Polyak, 2015). For instance, one study demonstrated that various clonal lineages in a case of colorectal cancer responded differently to treatment with chemotherapy (Kreso et al., 2013). Additionally, there is evidence that parallel evolution of various subclones within a tumor can lead to polyclonal resistance (Gerlinger et al., 2014). Additionally, intra-tumor heterogeneity makes it more difficult to precisely identify either histologically or genetically a tumor via a traditional biopsy (Tellez-Gabriel et al., 2016).

The implications of tumor heterogeneity in cancer evolution, clinical treatment, and tumoral spatial organization are not yet fully understood (Alizadeh et al., 2015), but scNGS provides a mechanism for beginning to unravel these relationships. Although heterogeneity makes the histological and genetic identity of a tumor more ambiguous, if the mechanisms driving heterogeneity are further elucidated, they may lead to a better understanding of carcinogenesis (Gay et al., 2016). Moreover, data gathered from single-cell sequencing may help to clarify the methods of cancer progression and subclone resistance to chemotherapeutic treatment by sequencing both smaller transcripts and whole genomes in single cellular representatives of heterogeneous populations (Baslan and Hicks, 2017).

Interestingly, scNGS also has implications in lineage tracking in the development of differentiated tissues, as it may help to further clarify the developmental pathways involved in tissue differentiation (Kester and van Oudenaarden, 2018). As discussed, the nervous and immune systems are both well-studied examples of cellular systems that display cellular heterogeneity. For example, this technique can be used to study the central nervous system, and has the potential to not only molecularly classify various neurons or groups of neurons, but also to further study the molecular mechanisms behind, and possible therapies for, neurological diseases (Ofengeim et al., 2017). Indeed, this application can also be utilized to type sperm and oocytes, allowing for the confirmation and subsequent study of recombination events and polymorphisms in these haploids (Zhang et al., 1992).

## Design of scNgs Experiments

One of the key questions in planning the methodology of a single-cell study is how many cells to sequence. Sequencing more cells enables a greater representation of the cells in a population, giving a more accurate model of the diversity of subclones. The number of single-cells sequenced in a study has scaled exponentially with the development of new technologies. In 2009, for example, only one cell could be sequenced at a time. By 2017, however, the technology has advanced enough to permit the analysis of hundreds of thousands of cells at once (Svensson et al., 2018) and the possibility to generate exobytes and even yottabytes of data in the future.

**FIGURE 1:**
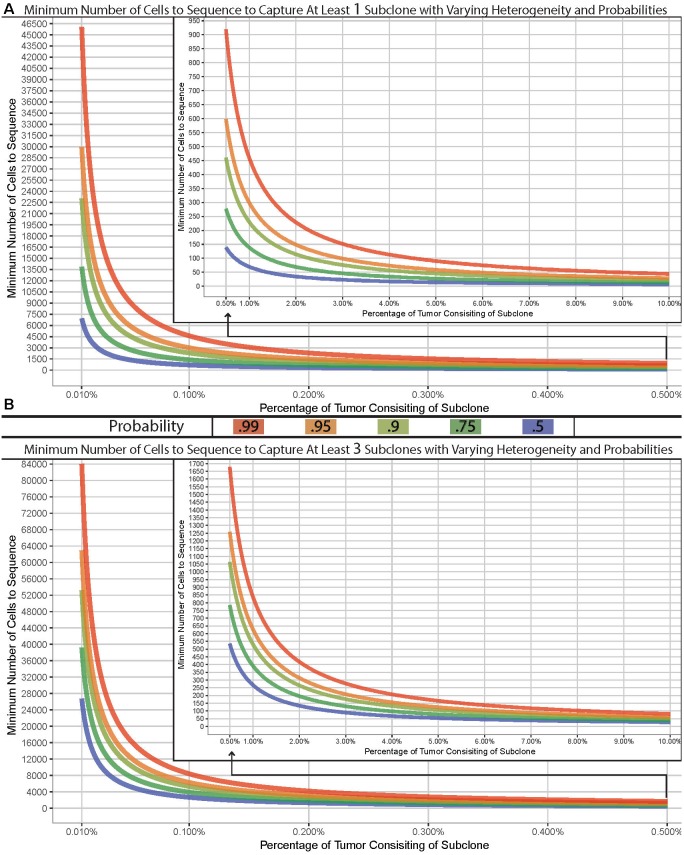
Model of cells required for detection of variants. Minimum number of cells to sample to capture at least one **(A)** or three **(B)** subclone with varying probabilities (lines) across varying concentrations in a tissue with 1 billion cells. Hypergeometric calculations were done using R’s phyper() function with lower.tail = F and *q* = 0 **(A)** or two **(B)** across varying sample sizes and clonal frequencies such that m+n = 1,000,000,000.

Many complexities exist with scNGS analyses and need to be carefully considered. Other work have covered the specific differences, benefits, and drawbacks between the various scNGS protocols (Kanter and Kalisky, 2015; Clark et al., 2016; Haque et al., 2017; Liang and Fu, 2017). Previous data have shown that the best scNGS technology should be used for a given hypothesis, in tandem with a proper experimental design for the number of cells. Due to this, the required number of cells necessary to address a given question or tissue model will largely vary depending on the overall hypothesis. However, the question of “how many cells should I sequence” can be simplified to how many cells do you need to sample in order to capture at least one subclonal cell. The chance of sampling a subclone from a tissue of interest depending on the subclonal prevalence, the size of the tissue, and the size of the sample. Therefore, this question can be modeled using the hypergeometric distribution with varying degrees of probability (Figure 1A). It is common within sc-NGS analysis to require multiple cells to contain a given phenotype, and therefore may be more appropriate to ask the question of “how many cells should I sample to capture at least three subclonal cells” (Figure 1B).

We have built a model to demonstrate the number of cells required for a sampling design can widely vary. As an example, if the goal was to sample a tissue which has 1 billion cells for a previously undefined stem-cell which exist at a population of 0.01%, you would have a 99% chance of sampling at least one stem-cell if you analyzed approximately 46,000 cells. However, to truly characterize and identify this subclonal population or to detect a lower threshold, the number of cells required could easily reach, or even surpass, 100,000 depending on tissue size (Figure 1B). Given the recent advances in scNGS and decreases in costs, this is now possible to do. Such a design – while completely impossible 5 years ago – should be strongly considered when designing experimentations today.

## The Future of Single-Cell Analyses

While single-cell sequencing has many advantages, it certainly is not a perfect technique. There are many different techniques for obtaining single-cell sequencing data and single-cell whole genome sequencing (sc-WGS), and each of these methods presents its own unique strengths and weaknesses. Multiple displacement amplification (MDA) and other PCR-based sequencing techniques often experience significant amplification bias (de Bourcy et al., 2014; Ahsanuddin et al., 2017). This could lead to incorrect interpretation of the prevalence and diversity of certain genes. Nonetheless, thanks to the breakthroughs in scNGS, the long-sought goal of sequencing of single cells is possible. This has created significant opportunities for advancement in the study of heterogeneity, especially as it applies to cancer. While it may be necessary to sample thousands or even millions of cells to encounter a unique subclone at low prevalence within a large tissue, sequencing continues to get cheaper, and thus scNGS will continue to open up many new research directions into the mechanisms of heterogeneity study variation on cell-by-cell resolution.

## Author Contributions

CM and SG conceived and designed the study. CM, SG, and MM analyzed the data. SG, MM, EA, AM, and CM wrote the paper. All authors, reviewed, edited, and approved the manuscript.

## Conflict of Interest Statement

The authors declare that the research was conducted in the absence of any commercial or financial relationships that could be construed as a potential conflict of interest.
